# Cellular Membranes, a Versatile Adaptive Composite Material

**DOI:** 10.3389/fcell.2020.00684

**Published:** 2020-08-05

**Authors:** Lucas Lamparter, Milos Galic

**Affiliations:** ^1^Institute of Medical Physics and Biophysics, Faculty of Medicine, University of Münster, Münster, Germany; ^2^Cells in Motion Interfaculty Centre, University of Müenster, Münster, Germany

**Keywords:** composite material, adaptive material, lipid bilayer, plasma membrane, cell cortex

## Abstract

Cellular membranes belong to the most vital yet least understood biomaterials of live matter. For instance, its biomechanical requirements substantially vary across species and subcellular sites, raising the question how membranes manage to adjust to such dramatic changes. Central to its adaptability at the cell surface is the interplay between the plasma membrane and the adjacent cell cortex, forming an adaptive composite material that dynamically adjusts its mechanical properties. Using a hypothetical composite material, we identify core challenges, and discuss how cellular membranes solved these tasks. We further muse how pathological changes in material properties affect membrane mechanics and cell function, before closing with open questions and future challenges arising when studying cellular membranes.

## Introduction

The cellular membrane is one of the most vital and complex biomaterials in live matter. It is therefore not surprising that it has over the last decades been the subject of extensive studies, giving insights into a fascinating world of regulatory processes that control localization and activity of proteins and lipids in cellular membranes (Singer and Nicolson, 1972; Edidin et al., 1991; Eggeling et al., 2009). These studies established that biomechanical requirements vary across species and subcellular sites, indicative of tremendous diversity and adaptability (Vasanji et al., 2004; van Meer et al., 2008). Yet, despite substantial advancements over the last few decades, we are still lacking a comprehensive understanding of membrane mechanics at the cell surface.

Individual lipids spontaneously self-assemble into a bilayer that is then subject to thermal fluctuations (Faucon et al., 1989), changes in local lipid composition (Estep et al., 1979), and sustained membrane deformations (Baumgart et al., 2003). Besides lipids, cellular membranes further contain proteins that not only influence the local lipid environment (Contreras et al., 2011; Laganowsky et al., 2014) and mechanical membrane properties (Shi et al., 2018), but also render the membrane transmissive to incoming stimuli. These signals can alter, among many others things, membrane potential (Nernst, 1889), shape (Chen et al., 2016; Graber et al., 2017) and lipid composition (Sugimoto et al., 1984). Importantly, signal-induced changes also affect localization and activity of membrane-bound (Carpenter et al., 1978) and cytosolic (Hancock et al., 1989) proteins. One principal target for such signals is the membrane-associated cortical cytoskeleton (Svitkina, 2020). Possibly the most striking of its many features is the ability of the cytoskeleton to generate mechanical push and pull forces (Peskin et al., 1993; Huxley and Simmons, 1971). Hence, by coupling membranes to the adjacent cell cortex yields a composite material that is equally able to sense and respond to external stimuli.

In this review, we will explore the intricate mechanical properties of this adaptive composite material. Readers interested in learning more on signaling across cellular membranes or cytoskeletal mechanics, we refer to the many excellent reviews written elsewhere (Huber et al., 2013; Lundberg and Borner, 2019; Svitkina, 2020). We begin by defining core mechanical terms, before investigating the material properties of the cell membrane. Next, we show how these properties are affected by its manifold interaction with the cell cortex under physiological and pathophysiological conditions. We conclude by pointing out future challenges toward understanding the mechanical properties of this exciting biomaterial.

## Fundamental Cell Mechanics

The plasma membrane and the cortical cytoskeleton jointly form a composite material with unique biomechanical features (Nicolson, 2014). To fully appreciate this elaborate structure, we first need to introduce a number of terms that will repeatedly show up over the following pages. Let’s begin by answering what a composite material actually is. This term – originating from manufacturing engineering – depicts two or more connected materials, where the resulting compound exhibits properties that neither of the individual components by itself possesses (Westkämper and Warnecke, 2011). The material properties of the composite are a function of the individual material properties of its components, their geometry and its interface (i.e., the way those materials are connected). Therefore, by changing one of those parameters the overall properties of the composite will be affected. If this happens by design, for instance in response to a stimulus, the material is referred to as an adaptive composite material. For biological membranes, the main components of this composite material are the plasma membrane (PM) that encapsulates the cell, the cell cortex lining the cytosolic site of the PM, and the membrane-cortex-attachment (MCA) that describes the interface connecting PM and cell cortex. In the following, we refer to the composite material emerging from the combination of PM, MCA and cell cortex as “cell surface.”

**FIGURE 1:**
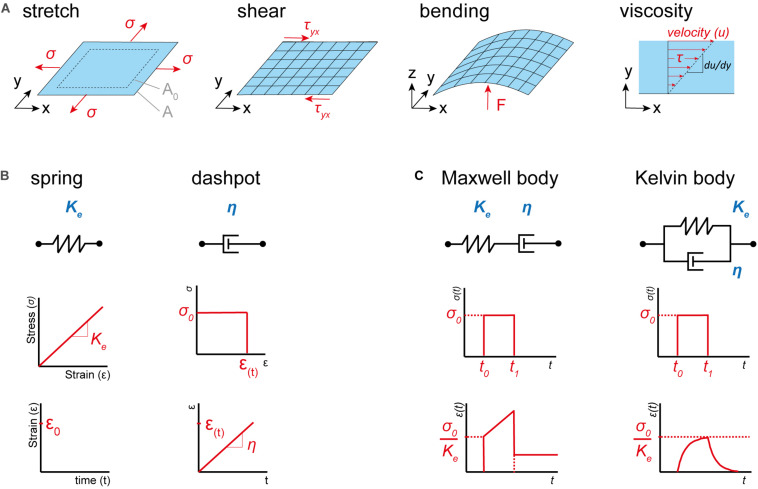
Basic material properties and deformation types. **(A)** From left to right, membrane exposed to uniform extensional stress σ, which is acting normal to its edges, causes an increase of the area (from A_0_ to A). Membrane exposed to forces acting parallel to its surface inducing shear stress τ_*yx*_. Membrane exposed to a point load (F) acting perpendicular to its surface, which is inducing curvature and therefore a bending moment. Viscosity η, the resistance of a fluid against shear stress τ given at a specific shear velocity $\frac{d\,u}{d\,y}$. **(B)** Basic properties of a purely elastic (spring, left) and viscous (dashpot, right) material. A purely elastic material always returns to its original state after deformation and exhibits a linear relationship between stress and strain, while a purely viscous material exhibits plastic (permanent) deformations and a linear relationship between stress and shear velocity. **(C)** Models of viscoelastic materials. Maxwell body (left) consisting of a dashpot and a spring in series displays, like a viscoelastic fluid, permanent deformation after the load is removed. Kelvin body (right) returns, like a viscoelastic solid, to its original state when the load is removed. Images adapted from Seidel et al. (2009) and Kamm and Grodzinsky (2015).

Having introduced the basic terms, we next need to recapitulate the essential material properties. For small strains, any membrane deformation can be regarded as the superposition of three basic types of deformation: pure stretch, shear and bending (Figure 1A). A membrane subject to a force acting normal to its edges will experience an extensional stress and an area stretch. The resistance against this stretch is called the stretch modulus *K*_*e*_. Similarly, a membrane that is subject to forces acting parallel to its edges will experience shear stress, whereby the resistance against the shear deformation is given by the shear modulus *K*_*s*_. While stretch and shear modulus relate strain to a certain stress, the bending stiffness *K*_*B*_ relates a curvature to a bending moment (i.e., the energy necessary to bend the membrane to its current state). The deformations described so far are purely elastic, and therefore not time dependent. However, biomaterials often exhibit fluid-like behavior, including time dependent plastic deformation. Therefore, we need to introduce a fourth material property, the viscosity η (Figure 1A, right). It depicts the resistance of a fluid against shear stress at a specific shear velocity $\frac{d\,u}{d\,y}$. In contrast to the shear modulus, the viscosity does not relate stress to strain, but to the rate of deformation. By changing one or a combination of these four essential material properties mechanical adaptation can be achieved. Readers interested in learning more about this topic, we refer to work written elsewhere (Kamm and Grodzinsky, 2015).

Finally, let’s recapitulate the types of deformations a material can experience. The properties of a material determine its response to a mechanical stimulus. In its most basic form, such a response can be purely elastic or viscous. An elastic material returns to its original shape upon distortion, while a viscous material stays distorted after the load is released. This behavior can be envisioned as a spring (elastic) or dashpot (viscous), respectively (Figure 1B). However, many materials display a viscoelastic response, where viscous and elastic properties are coupled. Basic models for such a behavior are the Maxwell and the Kelvin body (Figure 1C). In a Maxwell body, elastic and viscous elements are paired in series, thus leading to an elastic response for fast distortions, while slowly applied load is captured in a plastic manner. In contrast, the Kelvin body displays elastic and viscous elements in parallel. Hence, under stress the deformation will over time approach the strain response of the elastic component, whereby it is slowed down by the viscous element. Consequently, the Maxwell body is capable of plastic deformation (i.e., viscoelastic fluid), while the Kelvin body will always return to its original shape when the load is released (i.e., viscoelastic solid). As above, we refer readers interested in this topic to work written elsewhere (Meyers and Chawla, 2010).

## Engineering a Hypothetical Biomimetic Membrane

Inspired by the famous quote “*what I cannot create, I do not understand*” from Richard Feynman, we will attempt on the following pages to conceive a hypothetical biomimetic membrane with the following features: It should reliably separates internal from external space. The boundary should further be flexible, tunable, and able to transiently change its material properties in response to incoming signals. For simplicity and didactic reasons, we designed this as an engineering challenge (Supplementary Figure S1 and Supplementary Material), sequentially expanding the number of requirements (i.e., requested functions) of the hypothetical material. In each section, we begin by musing how such a biomimetic structure could be designed, before exploring in detail how this has been accomplished in biological systems.

### The Lipid Bilayer, the Core Module of Cellular Membranes

The most fundamental function of our biomimetic material is to reliably separate the interior from the external environment. To achieve such a continuous barrier in an aqueous solution, a biomimetic material must be able to oppose differences in osmotic and hydrostatic pressure without leakage. Ideally, the material would spontaneously assemble, thus limiting the need for energy and control systems.

**FIGURE 2:**
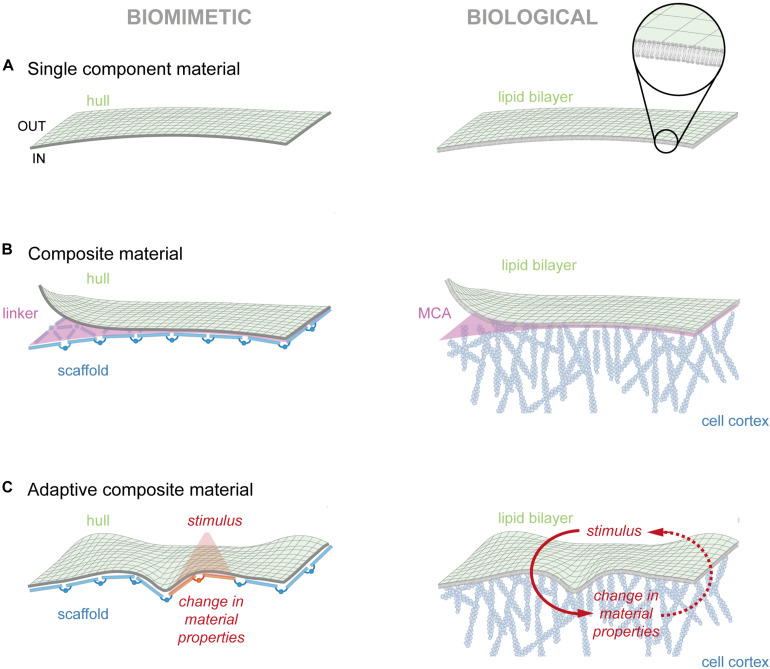
Modules of cellular membrane. **(A)** To the left, hypothetical biomimetic membrane that separates the inside from outside. To the right, the lipid bilayer presents the outermost structure of the cell membrane. Magnification depicts the 5–7 nm thick bilayer composed of individual lipids. **(B)** To the left, hypothetical composite material composed of a flexible hull associated to a rigid scaffold. To the right, the plasma membrane is tightly coupled to the cell cortex, creating a composite material. Individual actin proteins assemble into filaments that are facing the cell membrane with the growing end. **(C)** To the left, hypothetical adaptive composite material that changes material properties upon an external stimulus (red). To the right, the membrane/cortex continuum readily responds to external stimuli (red), reminiscent of an adaptive composite material. Note that changes in actin dynamics can also serve as signal (red, dotted line).

In cellular membranes, amphipathic lipids spontaneously self-assemble into a 5–7 nm thick lipid bilayer, with the hydrophobic carbohydrate chains facing toward the inside (Huang and Thompson, 1965). This occurs to minimize the free energy of water arising from continuous formation and disassembly of hydrogen bonds (Figure 2A, right). Pure lipid bilayers are reported to have a stretch modulus *K*_*e*_ in the range of 100 to 1000 mN/m (Waugh and Evans, 1979), with a value of ∼200 mN/m as most widely accepted (Rawicz et al., 2000). Considering a pure lipid bilayer with a possible areal stretch of 5% (Hallett et al., 1993), and neglecting thermal fluctuation (Evans and Rawicz, 1990), Eq. 2.5 in Box 1 gives us a rupture tension of 10 mN/m. This value is in accordance to published reports that measured a rupture tensions of 3 to 10 mN/m, depending on the lipid composition of the bilayer (Olbrich et al., 2000). Following Laplace’s law.

**Box 1. Physics at the Cell Surface.**

In the following, we consider the cell surface as a plate or shell encapsulating the cytoplasm, following the description by Kamm and Grodzinsky (2015). In general, any deformation can be envisioned as the superposition of three fundamental types of deformation: stretch, bending and shear. Surface tension is defined as the derivation of energy with respect to area, meaning the energy needed to stretch the plate by a certain amount is given by.

2.1 $$N=\sigma \,h=\frac{E\,h}{1-\nu }\,\varepsilon =\frac{E\,h}{2\,\left(1-\nu \right)}\,\frac{\Delta \,A}{A_{0}}=K_{e}\,\frac{\Delta \,A}{A_{0}},$$

where σ depicts the uniform normal stress, N the in plane tension in force per unit length (N/m), *E* the elastic modulus (Young’s modulus), ν the Poisson ratio, h the plate thickness, ε the strain and *K*_*e*_ the area stretch modulus defined as *K*_*e*_ = $\frac{E\,h}{2\,\left(1-\nu \right)}$. The energy needed to bend a plate of the thickness h in one dimension given by

2.2 $$M_{x}=E_{b\,e\,n\,d}=-\frac{E\,h^{3}}{12\,\left(1-\nu^{2}\right)}\,\frac{\delta^{2}\,u_{z}}{\delta \,x^{2}}=-K_{B}\,\frac{\delta^{2}\,u_{z}}{\delta \,x^{2}},$$

where $\frac{\delta^{2}\,u_{z}}{\delta \,x^{2}}$ depicts the deflection of the plate in z direction and *K*_*B*_ the bending stiffness defined as $K_{B}=\frac{E\,h^{3}}{12\,\left(1-\nu^{2}\right)}$. Elastic materials exhibit resistance against shear deformation arising when the lateral surfaces of the plate are exposed to two different surface tensions. This resistance is given by the shear modulus *K*_*s*_, with the unit N/m, relating the arising shear force per unit length *N*_*xy*_ to the amount of shear deformation, the strain ε_*xy*_. The shear force per unit length is the product of the shear stress τ_*xy*_ times the plate thickness h, which can also be described by the shear modulus *G* and the shear deformation ε_*xy*_. Corresponding to Eq. 2.2 the shear modulus can be expressed by Young’s modulus *E* and the Poisson ratio υ:

2.3 $$\sigma_{x\,y}=\tau_{x\,y}\,h=2\,G\,\varepsilon_{x\,y}=\frac{E}{1+\nu }\,\varepsilon_{x\,y}=K_{s}\,\varepsilon_{x\,y},$$

Considering the cells surrounding, we must apply for thermal fluctuation by adding a random force term *ξ*(*t*) and, for the damping of membrane movement by the extracellular fluid, we add a term in form of $\eta_{e}\,\frac{\delta \,u_{z}}{\delta \,\left(t\right)}$ with the effective viscosity of the surrounding fluid η_*e*_ dissipating its energy. Adding thermal fluctuation and energy dissipation due to external fluid damping, neglecting shear stress, yields

2.4 $$\Delta \,p+\xi \,\left(t\right)=K_{B}\,\left(\frac{\delta^{4}\,u_{z}}{\delta \,x^{4}}+2\,\frac{\delta^{4}\,u_{z}}{\delta \,x^{2}\,\delta \,y^{2}}+\frac{\delta^{4}\,u_{z}}{\delta \,y^{4}}\right)-N\,\left(\frac{\delta^{2}\,u_{z}}{\delta \,x^{2}}+\frac{\delta^{2}\,u_{z}}{\delta \,y^{2}}\right)+\eta_{e}\,\frac{\delta \,u_{z}}{\delta \,t},$$

which is giving us a more complete picture of the cell membrane behavior. Note that the principle of superposition is only valid for small strains, where linearization is appropriate (e.g., small discretization of time and space). Neglecting all terms except of pressure and surface tension – as it can be appropriate for small curvatures, Eq. 2.4 gives rise to the famous Laplace equation.

2.5 $$\Delta \,p=-N\,\left(\frac{\delta^{2}\,u_{z}}{\delta \,x^{2}}+\frac{\delta^{2}\,u_{z}}{\delta \,y^{2}}\right)≅N\,\left(\frac{1}{R_{1}}+\frac{1}{R_{2}}\right).$$

Biomaterials often exhibit viscoelastic properties. Therefore, we also have to consider the materials viscosity η. In contrast to the elastic modulus *E*, the viscosity relates the stress not to the deformation of the material but to the rate of change of the deformation $\frac{d\,\varepsilon \,\left(t\right)}{d\,t}$. One way to describe the properties of a viscoelastic solid is the Kelvin-Voigt model (see Figure 2C), which can be envisioned by an elastic spring and a viscous damper connected in parallel. The elastic properties of the material are given by the elastic modulus E, and the viscous properties by the viscosity η. The experienced stress σ is then given by

2.6 $$\sigma \,\left(t\right)=E\,\varepsilon \,\left(t\right)+\eta \,\frac{d\,\varepsilon \,\left(t\right)}{d\,t}.$$

However, the cell surface exhibits the ability of plastic deformation and therefore behaves more like a fluid. Regarding the cell cortex this property arises from the turnover rate of the linking proteins. In contrast to a viscoelastic solid, a viscoelastic fluid can be modeled by a Maxwell body, which can be envisioned by an elastic spring and viscous damper connected in series (see Figure 2C). The experienced stress σ is here given by

2.7 $$\sigma \,\left(\text{t}\right)+\frac{\eta }{E}\,\frac{\sigma \,\left(\text{t}\right)}{\text{dt}}=\eta \,\frac{d\,\varepsilon \,\left(t\right)}{d\,t}.$$

1.1 $$\Delta \,p=\frac{2\,N}{R},$$

where *N* depicts the surface tension and *R* the principal radius of curvature, we find that the PM can therefore withstand a pressure difference Δ*p* of up to 0.2 bar or 20 kPa assuming a radius of 1 μm before ripping, a remarkable performance given its thickness of only 5–7 nm.

With respect to the material properties mentioned at the beginning of the section, the lipid bilayer already provides excellent stretch resistance. However, it is readily deformed in the presence of forces within the plane (i.e., low shear resistance *K*_*s*_) or perpendicular to it (i.e., low bending rigidity *K*_*B*_). Hence, to strengthen the structural integrity of the boundary, and render it tunable, additional support is required.

### The Membrane-Cortex Interface Forms a Composite Material

For our hypothetical biomimetic material, the mechanical properties of the hull could be further improved by combining it with a self-assembling scaffold that binds with high affinity to the flexible hull to form a tri-partite composite: hull, supporting scaffold and the connecting glue (Figure 2B, left). As one can easily imagine, size, density and rigidity of the scaffold units will directly affect the material properties of the composite. However, these properties equally rely on the assembly pattern of the scaffold along the hull. For instance, scaffolds formed from elongated structures that align along a pre-defined axis will yield polarized (i.e., different) bending moduli along the principal curvatures. In contrast, an omnidirectional assembly of the same structures will produce isotropic mechanical properties. Besides the form of the supporting structure, the spacing between individual subunits also influences the bending stiffness (Naghieh et al., 2018). One such example is agarose gel, which exhibits substantially higher resistance to shear stresses than its primary component water. Hence, by wisely designing the geometry of a supporting structure, or the composition of matrix particles, materials with very diverse mechanical properties may emerge.

A lipid bilayer substantially differs from the cell surface, which is not only stiffer but also displays slower lateral diffusion (Edidin et al., 1991; Shi et al., 2018). This clearly posits the presence of an additional supportive material that interacts with the lipid bilayer. In cells, this composite material is formed through the interaction of the plasma membrane with the cell cortex (Figure 2B, right). The cell cortex consist of straight and branched actin filaments that are crosslinked among each other (Mullins et al., 1998), forming a 100–200 nm thick layer at the inner side of the PM (Chugh and Paluch, 2018). In addition to actin, the cell cortex contains, to a lesser degree, intermediate filaments and microtubules (Koning et al., 2008; Singh et al., 2018), providing the ability to exert mechanical forces and change local material properties. Membrane association of the cell cortex is achieved through a specialized protein group, the FERM domain proteins (named after its founding members 4.1 protein, ezrin, radixin, and moesin), which simultaneously bind to lipids and proteins (Chishti et al., 1998). This dynamic interface, also termed membrane-cortex-attachment (Diz-Muñoz et al., 2010), transforms the two separate structures into one composite material. From Eq. 2.2 in Box 1, we learn that the energy to buckle a material scales with the thickness cubed. Consequentially, the energy to bend a 200 nm thick cell cortex is orders of magnitude higher than for a 5–7 nm thin lipid bilayer. Hence, the contribution of the lipid bilayer to the overall cell surface stiffness can be neglected for shape changes at cellular length scales. Intriguingly, the high frequency regime of the fluctuation spectrum of membrane fluctuation assays seems to depend to a large extent on the mechanical properties of the PM (Gárate et al., 2015), suggesting that the membrane is at least partly able to swing freely at small length-scales. Similar high fluctuation modes at the scale of dozens of nanometers were also observed in vesicles (Betz and Sykes, 2012). Thus, the bending stiffness of the cell surface (see Figure 1A) is likely dominated by the cell cortex on the cellular level and by a partly unattached PM on sub-micron length scales (Salbreux et al., 2012).

The connection from cell cortex to membrane also affects surface tension (Chugh et al., 2017). The cell membrane is attached to the cortex via linker proteins, which in turn are put under tension by the cortical surface tension *N*_*cor*_, yielding.

1.2 $$N_{c\,o\,r}\,\frac{2}{R}=\rho_{l\,i\,n\,k}\,F_{l\,i\,n\,k},$$

where ρ_*link*_ depicts the linker protein density and *F*_*link*_ the force applied by each linker. Using Laplace law (see also Box 1, Eq. 2.5), we find.

1.3 $$\Delta \,p=N_{P\,M}\,\frac{2}{R}+\rho_{l\,i\,n\,k}\,F_{l\,i\,n\,k}=\left(N_{P\,M}+N_{c\,o\,r}\right)\,\frac{2}{R},$$

which means that cell surface tension *N*_*cell*_ is the sum of membrane tension *N*_*PM*_ and cortical tension *N*_*cor*_ (Sens and Plastino, 2015). Let’s have a closer look at membrane tension. In a lipid bilayer, local gradients in membrane tension (e.g., induced by rapidly pulling on a tether) equalize over the rest of the membrane in a matter of milliseconds (Shi and Baumgart, 2015), which is reminiscent of a fluid lacking shear stress. This, however, changes as soon as the PM is attached to the cellular cortex. Roughly one quarter of the total PM area is occupied by transmembrane proteins, half of which are connected to the cellular cortex (Bussell et al., 1995). These proteins, if attached to the cell cortex, serve as obstacles that limit membrane flow (*u*).

1.4 $$u=\frac{k}{\eta_{l\,i\,p\,i\,d}}\,\nabla N,$$

with Darcy permeability *k* of a porous medium (i.e., the array of obstacles) and lipid viscosity η_*lipid*_ (Shi et al., 2018). The lateral permeability *k* can be described as a function of the area fraction ϕ occupied by the integral proteins and their radius *a* (Happel, 1959).

1.5 $$k\approx -\frac{a^{2}\,\left(1+\ln\left(\phi \right)\right)}{8\,\phi }.$$

Hence, if confined into two dimensions, the flow resistance decreases logarithmically with the distance to the obstacle (rather than exponential). Since the logarithmic function diverges for large distances, the effect will be noticeable no matter how far away from the obstacle we evaluate the system - a phenomenon called the Stokes paradox. The same study further noted that the surface tension of a PM attached to the cell cortex propagate over time in a diffusive manner, given by.

1.6 $$\frac{\delta \,N}{\delta \,t}=\frac{E\,k}{\eta_{l\,i\,p\,i\,d}}\,\nabla^{2}N,$$

where *E* depicts the elastic modulus of the PM and η_*lipid*_ the viscosity of the lipids (i.e., the fluid’s viscosity flowing through these obstacles). Note that Eq. 1.6 exhibits a striking similarity to Fick’s second law of diffusion. In their elegant study, Shi and Cohen defined a tension diffusion coefficient *D*_*N*_

1.7 $$D_{N}=\frac{E\,k}{\eta_{l\,i\,p\,i\,d}},$$

yielding

1.8 $$\frac{\delta \,N}{\delta \,t}=D_{N}\,\nabla^{2}N.$$

Consequently, the cortex bound integral proteins cause local and long lived gradients of membrane tension. It is plausible to envision that cells may locally alter the tension diffusion coefficient through MCA-induced changes in number and position of transmembrane obstacles (i.e., change ϕ). Consistently, recent work showed a positive correlation between membrane-proximal actin density and membrane tension (Bisaria et al., 2020). If and how membrane tension gradients influence cortical tension *N*_*cor*_, and in consequence cell tension *N*_*cell*_, remains elusive.

Finally, let’s consider membrane viscosity. PM viscosity differs from the viscosity of a pure lipid bilayer due to the embedded integral membrane proteins (IMPs). For a suspension with no interparticle interactions (i.e., low concentration), the effective viscosity of such a suspension can be approximated by.

1.9 $$\mu_{e}=\mu_{0}\,\left(1+B\,\phi \right),$$

where μ_*0*_ is the viscosity of the suspending liquid, ϕ volume fraction of the embedded particles and *B* a coefficient depending on the particle shape (e.g., spheres, cylinders, disks) (Bird et al., 2007). Hence, it is possible that the attachment, and therefore immobilization of IMPs, may also affect PM viscosity. Despite the existence of some approximations on the effective viscosity (Larson and Higdon, 1986, 1987; Kolodziej, 1988), this complex topic remains unresolved and is beyond the scope of this review (see also Supplementary Material). Importantly, the sole plasma membrane exhibits viscous material properties. Attachment of to the PM to the cell cortex (i.e., the cell surface), however, displays a combination of viscous and elastic properties on the cellular length scale (Bausch et al., 1998). This means that on short time scales (i.e., ∼1 s) the cell surface responds like an elastic material, while it shows properties of a viscous material on longer time scales (i.e., 10–100 s). Considering that the cortex consist of interlinked actin filaments, which are subject to continuous turnover of cross-linking proteins and actin filaments, the cell cortex is able to perform plastic deformation reminiscent of a viscoelastic fluid. This behavior can, in its simplest form, be modeled as a Maxwell body (Begemann et al., 2019) described by Eq. 2.7 in Box 1 (see also Figure 1C).

In conclusion, our short survey demonstrates how interactions of membranes with the cell cortex alter the mechanical properties of the PM itself as well as of the cell surface. Yet, despite the versatility in material properties that can be achieved, the resulting composite material at this point still lacks the ability to readily respond to changes from the environment (i.e., adaptive response).

### The Cell Surface Is an Adaptive Composite Material

In the form described this far, the composite material will always thrive to adopt the minimal energy state. Strikingly, mechanical properties of membrane and cortex can be separately tuned with high spatial and temporal accuracy, providing the possibility to turn the passive composite into an active material. In our hypothetical composite material, such adaptivity could be achieved by rendering its components sensitive to a transient stimulus (e.g., light, temperature). The response to such a stimulus, which triggers local changes in one (or a combination) of the four essential material properties (Figure 1A), yields an adaptive composite material (Figure 2C, left).

Following, we will discuss how stimulus-induced transient changes in mechanical properties of membrane or cortex yield an adaptive composite material. We begin with tension. As mentioned above in Eq. 1.3, the tension of the cell surface is the sum of the PM tension and cortex tension, and therefore a function of the density of linker proteins ρ_*link*_ and the stretch moduli of the plasma membrane $K_{e}^{P\,M}$ and cell cortex $K_{e}^{c\,o\,r}$. These parameters, in turn, rely on the respective elastic moduli *E*, thickness *h*, and the Poisson ratio ν (Eq. 2.1, Box 1). Hence, signal-induced changes to any of those properties will necessarily influence the cells surface stretch modulus $K_{e}^{c\,e\,l\,l}$. The stretch resistance of the lipid bilayer $K_{e}^{P\,M}$, for instance, arises from energy penalty caused by exposure of the hydrophobic core to the surrounding aqueous solution. It could therefore, in theory, be tuned by changing the membrane lipid composition, which may also effect the membrane thickness h. However, with a modulus of ∼200 mN/m (Rawicz et al., 2000), the lipid bilayer is not a strongly extendable structure, and thus not well suited. Note that most values in literature describe the apparent stretch modulus of the PM, which integrate large scale changes in membrane area originating from membrane undulation, membrane folds, vesicle fusion, the smoothening of membrane fluctuations and other sources (Le Roux et al., 2019). Complementing membrane-based tension, cortex-based tension can be modulated by signal-dependent changes in actin cross-linker density, the MCA (i.e., density of PM-cortex linker proteins ρ_*link*_) and cortical myosin (Stewart et al., 2011). In particular, the latter renders the composite material suitable to adaptation on physiological timescales (i.e., order of seconds), whereby lipid composition as well as the cortex geometry (e.g., mesh size) will affect the respective elastic moduli.

#### Bending Stiffness

As membrane and cortical stiffness critically depend on the length scale, we will consider their contribution separately. The bending stiffness of the PM, which is relevant in the sub-micron regime, depends on the composition of the lipid bilayer (Helfrich, 1973). By changing length and saturation of individual carbohydrate chains, the lipid bilayer thickness, and thereby the bending energy, can be modulated (see Eq. 2.2 in Box 1). Adding hydrophobic heads with varying size further renders lipids curvature-sensitive, while differences in charge will influence protein-lipid interactions with peripheral cytosolic proteins (Ebrahimkutty and Galic, 2019; Bassereau et al., 2018). The bending stiffness of the cell cortex, which is relevant in the μm regime, can be influenced by changes to the actin mesh size (depicted by the density of cortex linker proteins) or the thickness of the cortex itself. In addition, signals that change the elastic moduli of both materials (discussed above) will also influence their respective bending rigidities $K_{B}^{P\,M}$ and $K_{B}^{c\,o\,r}$ (see Eq. 2.2 in Box 1).

#### Shear Resistance and Viscosity

Pure lipid bilayers behave like a fluid void of shear stress (Mofrad and Kamm, 2006). This is not always the case in cellular membranes (Shi et al., 2018). From Eq. 2.3 in Box 1, we deduce that all cellular stimuli that influence the shear modulus (and therefore Young’s modulus *E* as well as the Poisson’s ratio υ, as discussed above) will also affect the elastic shear stress response of both materials, and therefore also of the cell surface. As the cell cortex as well as the attached PM both exhibit viscoelastic properties, we further have to consider the control variables of the respective viscosities. In case of the cortex, the dominating control variable (i.e., target for adaptive stimuli) are the relative turnover rates of actin and its cross-linkers, as well as the relative mesh size of the cell cortex (Boulbitch et al., 2000; Kasas et al., 2005). Regarding the PM, and recalling the description of Stokes paradox from the previous paragraph, viscosity of the PM could be modulated by signal-induced changes to the total concentration of integral proteins, its spatial distribution (e.g., clustering), and the fraction of cortex bound units.

The findings summarized in this section highlight the manifold strategies for signal-induced changes in individual material properties that render the cell surface an adaptive composite material. How these material properties are changed under pathophysiological conditions is the topic of the following chapter.

## Faulty Material Properties Cause Disease

In any engineered structure, incorporation of faulty materials yields catastrophic consequences for the over-all stability of the object. In cells, such faulty materials manifest as disease. Following, we survey disease arising from individual materials, the resulting composite and its adaptive features, respectively.

As described in the previous section, changes in relative lipid levels within the membrane may not only deplete components involved in signaling, but could also alter its material properties. Mounting evidence exist for changes in lipid balance [e.g., (cholesterol)↑, (sphingomyelin)↑ and (phosphatidylinositol)↓] during aging, but also in lipidosis such as Niemann-Pick disease types A and C (Karten et al., 2002; Arroyo et al., 2014), establishing how mechanical properties of the membrane may be altered in disease.

Intriguingly, faulty materials that lead to disease can also be found in cell cortex proteins. For instance, mutations in smooth muscle α-actin (*ACTA2*), which alter interactions between actin and myosin (Lu et al., 2015), cause vascular disease and stroke (Guo et al., 2009). Mutations in *ACTB*, the gene encoding cytoplasmic β-actin, change actin polymerization dynamics (Hundt et al., 2014), resulting in deafness, cancer and developmental disorders (Procaccio et al., 2006). Mutations in the gene encoding α-actinin-4 (*ACTN4*), an actin cross-linking protein, cause proteinuric kidney disease (Kaplan et al., 2000). Collectively, these few examples illustrate how changes in material properties of cell cortex components yield substantial physiological effects.

Disease can further be traced back to errors of the composite material. As described above, lipid composition determines rigidity and fluidity of membranes. Strikingly, some of these lipids form highly dynamic and heterogeneous membrane nanodomains (Head et al., 2014; Sechi and Wehland, 2000), which can be stabilized to form larger signaling platforms that are associated with actin filaments (Oliferenko et al., 1999; Galic et al., 2012; Begemann et al., 2019). As lipid availability or dynamics is changed in certain disease, the resulting mechanical properties of the membrane-cortex composite are likely altered in such conditions.

Finally, mutations can also affect the adaptability of the composite. One major family of disease originates from mutations in receptor proteins, which disturb signal-induced changes in material properties of the composite. One such example are G-protein coupled receptors (GPCRs), which alter among others the material properties of the cell cortex (Bhattacharya et al., 2004). Here, mutations cause aberrant signaling by disrupting surface expression (Ward et al., 2012), basal activity (Seifert and Wenzel-Seifert, 2002), or ligand binding (Venkatakrishnan et al., 2013). Furthermore, GPCRs directly interact with lipids, which means that changes in membrane composition or mutations in the lipid-recognition motifs can alter GPCR signaling by changing its stability, subcellular localization or activity (Keri and Barth, 2018; Pucadyil and Chattopadhyay, 2006). Importantly, errors in signal-induced changes in material properties can also be traced back to other receptor families (Jiang and Gonen, 2012; Fruman et al., 2017; Zakany et al., 2020), giving rise to an ever-growing family of membrane-related disease. A second major family of disease that influences the adaptability of the composite material relies on membrane curvature. Here, transiently deformed membrane sections trigger recruitment of curvature-sensitive proteins (Peter et al., 2004) and lipids (Wang et al., 2007; James et al., 2010). Notably, many of the more than 100 known curvature-sensing molecules identified in cells directly alter actin polymerization, thus changing mechanical forces applied to the PM. This dual ability of selected molecules to sense and modify actin-induced PM curvature is important, as it posits the core properties of an adaptive composite material. Consistently, defects of individual force-regulatory feedback loops (e.g., *OPHN1* and *SRGAP2*) result in very specific diseases (Billuart et al., 1998; Charrier et al., 2012).

While by far not complete, these selected examples illustrate how the material properties of individual components critically influence the overall mechanical state of the cell surface – both, in health and disease. How the mechanical properties of this versatile adaptive composite material are measured will be discussed in the next section.

## Measuring Material Properties in Cellular Membranes

The picture drawn this far depicts the importance of a functioning interplay between cortex, membrane and MCA. Considering its small scale, separating cell cortex from membranes in living cells is a challenging task (Diz-Muñoz et al., 2018). To gain insights into membrane tension, previous studies have taken advantage of pipets and optical tweezers to measure the force needed to extract a membrane tether from the cell surface (Raucher and Sheetz, 2000). Another approach relies on time-resolved membrane fluctuation spectroscopy (Betz and Sykes, 2012). Here, an optical tweezer is being used to measure membrane deformations rather than to pull tethers. In such measurements, the power spectral density of low-frequency fluctuations drops as tension increases (Betz and Sykes, 2012). Notably, fluctuation spectroscopy can also be used to gain insights into bending rigidity and viscosity of the material (Betz and Sykes, 2012). Quantitative measurements on membrane viscosity can also be acquired using high-throughput single molecule tracking or fluorescence correlation spectroscopy (Barbotin et al., 2019).

Yet, as mentioned above, changes in cortex bound protein spacing will affect membrane fluidity (Cohen and Shi, 2020). Hence, measurements from tether pulling and membrane fluctuation present a convolute of membrane and cell cortex properties (Shi et al., 2018). Thus, while each of these approaches allows exciting novel insights into the mechanical properties of cell membranes, it remains a challenging task to unequivocally dissect the contribution of the individual components of the composite material.

## Conclusion and Outlook

Seminal work one pure lipid bilayers, which are the topic of other reviews in this special issue, have substantially contributed to our current understanding of membrane mechanics. Yet recent studies argue that some cellular membranes should be considered an adaptive composite material (Shi et al., 2018; Cohen and Shi, 2020). In this review, we used a hypothetical biomimetic material to elucidate the astounding versatility of cellular membranes. We discussed how small changes in the mechanical properties of individual core modules allow the material to readily adapt its properties, thus fulfilling highly diverse functions, while relying on the same building materials. Strikingly, the composite nature of the cell surface not only allows cellular membranes to rapidly adjust its mechanical properties to the given situation, but also provides explanations to disease and some seemingly contradicting properties of the cell surface. One such example is the need for efficient transport of large objects across the cell cortex, while maintaining structural integrity at all times (Wollman and Meyer, 2012). As mentioned above, the lipid-cortex interface largely relies on non-covalent interactions. In consequence, the cytoskeleton continuously de- and reattaches from the lipid bilayer. These transient detachments not only open up the possibility to readily adjust interactions, but also the ability to transport and fuse vesicles (i.e., large objects) without permanent detachment of the plasma membrane from the underlying cortex.

Using the analogy of a hypothetical biomimetic material we realize that man-made materials still lag far behind its biological counterparts. It is encouraging, however, that this opportunity has been recognized, nucleating a rapidly growing research field (Amaral and Pasparakis, 2019; Chen and Li, 2020). Future advancements into bioinspired and biomimetic material will likely benefit from computational approaches (Sun et al., 2018; Schroer et al., 2020), which allow rapid advancement of theoretical and experimental data of membrane-MCA-cortex dynamics at an ever-increasing spatio-temporal resolution.

## Author Contributions

Both authors listed have made a substantial, direct and intellectual contribution to the work, and approved it for publication.

## Conflict of Interest

The authors declare that the research was conducted in the absence of any commercial or financial relationships that could be construed as a potential conflict of interest.
