# Supplementary material for: Cellular Membranes, a Versatile Adaptive Composite Material
Source: Front Cell Dev Biol. 2020 Aug 5;8:684. doi: 10.3389/fcell.2020.00684 (PMC7419611; doi:10.3389/fcell.2020.00684)
Supplement: Supplementary file 1 [file Data_Sheet_1.PDF]

Supplemental material for

Cellular membranes, a versatile adaptive composite material

Lucas Lamparter[1,2] and Milos Galic[1,2,*]

Content:

- Supplemental Text
- Supplemental References
- Supplemental Figures

SUPPLEMENTAL TEXT

The cell membrane, an engineering perspective.

To make the membrane properties more tangible, we will apply an engineering strategy. To that end, we adhere to the standard procedure for designing technical systems (Verein Deutscher Ingenieure, 1993), which divides this design challenge in four different phases (**Supplemental Fig. 1a**). Phase 1 defines the core requirements that should be met by the membrane. Phase 2 identifies functional units matching those requirements, and finds principal solutions for all units. Phase 3 divides the system in sub-modules and designs a structure, where each module represents one functional unit and the sum of all modules yields the overall design. Phase 4 executes the overall design.

Depending on the subcellular context, the core requirements of a cell surface may substantially differ. One strategy to achieve this versatility would be to separately define one functional unit for each requirement. In order to find principal solutions and design these modules, we envision the cell surface as a technical system. Every technical system (i.e. cell surface), can now be modeled as a black box that is transferring energy (e.g. ATP to mechanical forces), matter (e.g. transport of molecules) or information (e.g. receptors) (**Supplemental Fig. 1b**, top). Additionally, the structure of each black box can be described by the interrelation of physical (e.g. Van der Waals forces), geometrical (e.g. cylindrical bundled actin filaments) and material (e.g. stiffness) features (Weizsäcker, 1981). Hence, to design a biomimetic membrane using this top-down approach, all physical, geometrical and material features for all functional units and core modules need to be computed. With each requirement that is added, the system becomes more complex, which quickly renders this a daunting task.

How could we improve our design? Let's revisit Phase 3, and explore how the actual cell solved this challenge. We find that this is achieved by altering mainly two ingredients of the black box: the geometrical and material properties. These changes, in turn, force the interrelation of all ingredients into a different state, which is suitable to fulfill a completely different function, while using the same core modules (**Supplemental Fig. 1b**, bottom). At this point, we realize that the cell membrane must consist of an active adaptive composite material.

On the effective viscosity of the cell surface

Flow through a porous medium (i.e. array of obstacles), which is sufficiently slow to neglect inertial effects, can be described by the Brinkman equation (Brinkman, 1949)

$$\nabla p = \eta_{eff} \Delta \boldsymbol{u} - \frac{\eta_{fluid}}{k} \boldsymbol{u} \ , \tag{3.1}$$

where p is the pressure, u the velocity, η_{fluid} the fluid viscosity and η_{eff} the effective viscosity observed on the macroscopic level. Envisioning the plasma membrane as a porous medium, due to cortex-bound integral proteins, (Shi et al., 2018) used this equation as starting point to model tension propagation on living cell surfaces. Here, analogous to a pressure driven fluid, lipids flow from regions of low surface tension to those of high surface tension

$$\nabla N = -\eta_{eff} \Delta N + \frac{\eta_{lipid}}{k} N. \tag{3.2}$$

The effective viscosity η_{eff} in equation 3.2 therefore reflects the viscosity of the plasma membrane observed on the macroscopic level, while η_{lipid} depicts the sole lipid viscosity. Beside the lipid viscosity η_{lipid} and the permeability k (for which we can make reasonable guesses), we also require knowledge about the effective

viscosity η_{eff} of the medium in order to solve the Brinkman equation. Unfortunately, to date quantitative data towards η_{eff} remains very limited. For a suspension with non-interactive particles, the effective viscosity of the medium can be approximated by

$$\mu_e = \mu_0(1 + B\phi), \tag{3.3}$$

where μ_0 is the viscosity of the suspending liquid, ϕ volume fraction of the embedded particles and B a coefficient depending on the particle shape (e.g. spheres, cylinders, disks) (Bird et al. 2007). Referring to Einstein's equation, (Brinkman, 1949) suggested to approximate the effective viscosity η_{eff} of an highly porous medium consisting of immobile spheres as

$$\mu_e = \mu_0(1 + \frac{5}{2}\phi) , \tag{3.4}$$

where the quantity ϕ depicts the volume fraction of the particles. Although later studies confirmed the validity of equation 3.4 for media containing spherical obstacles (Lundgren, 1972; Freed and Muthukumar, 1978; Kim and Russel, 1985), the questions about the approximation of η_{eff} for other porous materials remains elusive, and η_{eff} is often simply approximated as $\eta_{eff} = \eta$ (Howells, 1974; Bussell et al., 1995; Shi et al., 2018). Intriguingly, attempts have been made to investigate the effective viscosity of a fluid flowing through a two dimensional array of cylindrical obstacles (Larson and Higdon, 1986, 1987; Kolodziej, 1988). In first approximation, this can be seen as a simplistic description of a plasma membrane with immobile integral proteins attached to the cortex. Like in equation 3.4, those authors find the effective viscosity η_{eff} to be dependent on the area fraction ϕ of the obstacles, thus strengthening the hypothesis that the effective viscosity of the PM indeed depends

on the MCA density. Readers interested in learning more about this exciting topic, we refer to the original literature referenced throughout the text.

SUPPLEMENTAL REFERENCES

Brinkman, H. C. (1949). A calculation of the viscous force exerted by a flowing fluid on a dense swarm of particles. *Appl. Sci. Res.* 1, 1045.

Bussell, S. J., Koch, D. L., and Hammer, D. A. (1995). Effect of hydrodynamic interactions on the diffusion of integral membrane proteins: tracer diffusion in organelle and reconstituted membranes. *Biophys J* 68, 1828–1835.

Freed, K. F., and Muthukumar, M. (1978). On the Stokes problem for a suspension of spheres at finite concentrations. *The Journal of Chemical Physics* 68, 2088–2096.

Howells, I. D. (1974). Drag due to the motion of a Newtonian fluid through a sparse random array of small fixed rigid objects. *J. Fluid Mech.* 64, 449–476.

Kim, S., and Russel, W. B. (1985). Modelling of porous media by renormalization of the Stokes equations. *J. Fluid Mech.* 154, 269–286.

Kolodziej, J. A. (1988). Influence of the porosity of a porous medium on the effective viscosity in Brinkman's filtration equation. *Acta Mechanica* 75, 241–254.

Larson, R. E., and Higdon, J. J. L. (1986). Microscopic flow near the surface of two-dimensional porous media. Part 1. Axial flow. *J. Fluid Mech.* 166, 449.

Larson, R. E., and Higdon, J. J. L. (1987). Microscopic flow near the surface of two-dimensional porous media. Part 2. Transverse flow. *J. Fluid Mech.* 178, 119–136.

Lundgren, T. S. (1972). Slow flow through stationary random beds and suspensions of spheres. *J. Fluid Mech.* 51, 273–299.

Shi, Z., Graber, Z. T., Baumgart, T., Stone, H. A., and Cohen, A. E. (2018). Cell Membranes Resist Flow. *Cell* 175, 1769-1779.e13.

Verein Deutscher Ingenieure (1993). *VDI 2221 Methodik zum Entwicklen und Konstruieren technischer Systeme und Produkte*. VDI Verlag.

Weizsäcker, C. F. (1981). *Einheit Der Natur*. [Place of publication not identified]: Hanser.

SUPPLEMENTAL FIGURES

Supplemental Figure 1. Designing an adaptive composite material. (a) Generalized design workflow. Individual phases (black), tasks (green) and results (green), and its interrelation, are highlighted in the respective colors. A core module (red) executes a function by changing energy, matter or information content of the system. **(b)** Simple modules carry one function (top), while adaptive core modules are suitable to change its output in dependence of the current state (bottom).

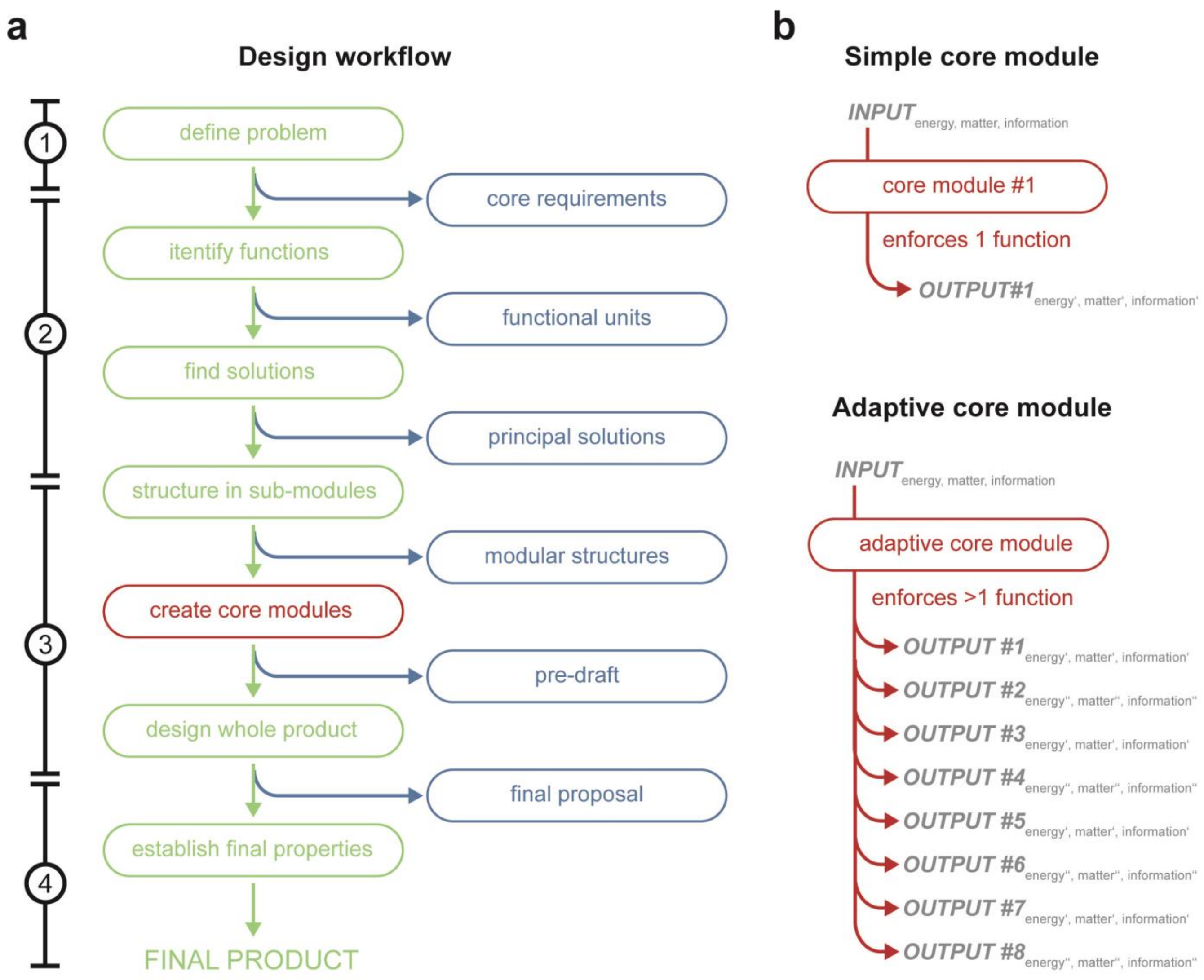

Suppl. Figure 1
Lamparter *et al.*
